# Therapeutic effects and central mechanism of acupuncture and moxibustion for treating functional dyspepsia: study protocol for an fMRI-based randomized controlled trial

**DOI:** 10.1186/s13063-022-06411-9

**Published:** 2022-06-06

**Authors:** Pan Zhang, Tao Yin, Yang-Ke Mao, Zhao-Xuan He, Sha Yang, Si-Qin Huang, Rui-Rui Sun, Fang Zeng

**Affiliations:** 1 Acupuncture and Tuina School/The Third Teaching Hospital, Chengdu University of Traditional Chinese Medicine, Chengdu, 610075 China; 2 Acupuncture and Brain Science Research Center, Chengdu University of Traditional Chinese Medicine, Chengdu, 610075 China; 3 Traditional Chinese Medicine College, Chongqing Medical University, No.1 Medical College Road, Yuzhong District, Chongqing, 400016 China

**Keywords:** Functional dyspepsia, Central mechanism, Acupuncture, Moxibustion, Functional magnetic resonance imaging

## Abstract

**Background:**

Functional dyspepsia (FD) is one of the most common functional gastrointestinal disorders, with a high prevalence and significant influence on the quality of life (QoL). Either acupuncture or moxibustion is effective for dyspepsia, which is confirmed by both ancient documents and modern research. However, the therapeutic advantage and underlying mechanism between acupuncture and moxibustion for FD remain unclear.

**Methods:**

This randomized controlled fMRI trial aims to (i) evaluate the therapeutic advantages of acupuncture and moxibustion treatment for FD, (ii) investigate the similarities and differences in cerebral activity elicited by acupuncture and moxibustion, and (iii) analyze the possible correlations between brain responses and clinical variables thus to explore the potential central mechanism of acupuncture and moxibustion for treating FD. Ninety-two FD patients will be randomly assigned to either the acupuncture group or the moxibustion group in a 1:1 ratio. Twenty sessions of acupuncture or moxibustion treatment over 4 weeks will be performed on each patient. The short form Leeds Dyspepsia Questionnaire, the Nepean Dyspepsia Index, etc., are used to evaluate the therapeutic effects. The heart rate variability will be analyzed to investigate the autonomic nerve function. Thirty-six FD patients in each group will be randomly selected for the fMRI scan to detect cerebral activity changes.

**Discussion:**

We expect the results will deepen our knowledge on the clinical value and underlying mechanism of acupuncture and moxibustion and provide a reference for a better selection of interventions for treating FD.

**Trial registration:**

Chinese Clinical Trial Registry (www.chictr.org.cn) ChiCTR2100049496. Registered on 2 August 2021

**Supplementary Information:**

The online version contains supplementary material available at 10.1186/s13063-022-06411-9.

## Background

Functional dyspepsia (FD), as one of the common functional gastrointestinal disorders (FGIDs), refers to a group of upper gastrointestinal syndromes, including epigastric pain, epigastric burning, meal-related fullness, and early satiation [1]. According to the Roma IV criteria, FD can be divided into three subgroups, the postprandial distress syndrome (PDS), the epigastric pain syndrome (EPS), and the overlap [2]. It was estimated that 80% of patients with dyspepsia are diagnosed as FD; what is worse, FD affects up to 16% of the individuals in the general population [3, 4]. Although FD is not a lethal disease, it significantly reduces patients’ quality of life (QoL) and impose an enormous socioeconomic burden on the healthcare financial system [5]. In the USA, the medical expense was estimated to be over $18 billion per year [6].

Acupuncture and moxibustion have been used for gastrointestinal symptoms for more than 2500 years in China and are increasingly accepted as the nonpharmacologic alternative therapies for FGIDs around the world [7–9]. Recent clinical studies have demonstrated that both acupuncture and moxibustion could effectively relieve symptoms and improve the QoL of FD patients [10, 11]. However, the advantages and the underlying mechanisms of acupuncture and moxibustion in the treatment of FD have not yet been fully elucidated.

In Roma IV criteria, FD is defined as a disorder of brain-gut interaction [12]. Therefore, exploring the pathological characteristics and therapeutic effect targets of FD from the perspective of brain activity arouses increasing attention in the past two decades. By neuroimaging techniques including PET and MRI, people found that FD patients have significant cerebral functional abnormalities and structural alterations, and the cerebral changes are related to their symptoms [13–17]. For example, our previous studies indicated that the anterior cingulate cortex (ACC), insula, thalamus, middle cingulate cortex (MCC), and cerebellum might be the key regions that were close to the severity of dyspepsia symptoms [18, 19]. Furthermore, studies have suggested that acupuncture treatment can effectively modulate the abnormal cerebral activities to improve dyspepsia symptoms and QoL, and moxibustion treatment also can elicit the cerebral signal changes in FGIDs patients [19–22]. These studies provide the possibility to explore the different therapeutic mechanisms between acupuncture and moxibustion from the central responses. Based on our previous studies, the present study is conducted to (i) evaluate the therapeutic advantages of acupuncture and moxibustion treatment for FD, (ii) investigate the similarities and differences in cerebral activity elicited by acupuncture and moxibustion, and (iii) analyze the possible correlations between the brain responses and clinical variables thus to explore the potential central mechanism of acupuncture and moxibustion respectively.

## Methods/design

### Design and setting

**Fig. 1:**
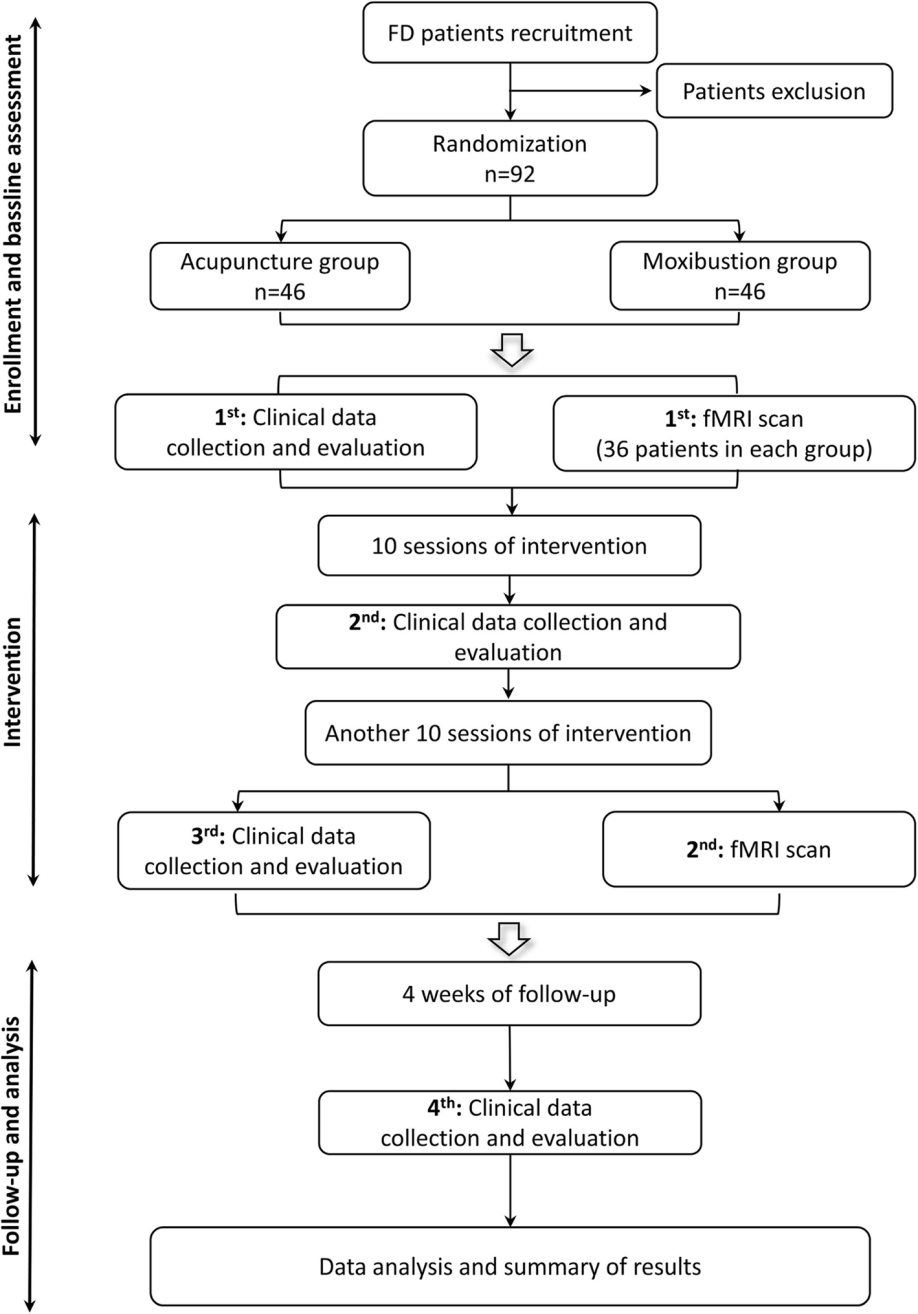
Schematic diagram. FD, functional dyspepsia; fMRI, functional magnetic resonance imaging

The present study is a single-center, parallel-controlled, equivalence, randomized clinical trial, and a total of ninety-two patients with FD will be recruited. These patients will be randomly allocated into either the acupuncture group or the moxibustion group with a 1:1 ratio. The study period includes a 1-week baseline, 4 weeks of treatment, and another 4 weeks of follow-up. Clinical measurements will be performed at the baseline, at the end of the treatment, and at the end of follow-up, respectively. Neuroimaging scans will be performed at the baseline and the end of the treatment. More details of the study design are shown in Fig. 1 and Table 1.

**Table 1:** Schedule of enrollment, interventions, fMRI scan, and assessment

Study period week	Baseline		Treatment period				Follow-up			
Time point	^**a**^***0-wk.***	***1-wk.***	***2-wk.***	***3-wk.***	***4-wk.***	***5-wk.***	***6-wk.***	***7-wk.***	***8-wk.***	***9-wk.***
**Enrolment**										
Patient screen										
Informed consent	★									
Physical examination		★								
Laboratory test		★								
Randomization		★								
**Interventions**										
Acupuncture group			★	★	★	★				
Moxibustion group			★	★	★	★				
**fMRI scan**										
Acupuncture group		★				★				
Moxibustion group		★				★				
**Assessment**										
SF-LDQ		★				★				★
NDI		★		★		★				★
SID		★		★		★				★
SAS and SDS		★		★		★				★
HRV		★				★				
*Deqi* Sensation			★			★				
Safety monitoring	★	★	★	★	★	★	★	★	★	★

^a^The screening visits

*wk.* week, *SF-LDQ* short form Leeds Dyspepsia Questionnaire, *NDI* Nepean Dyspepsia Index, *SID* Severity Index of Dyspepsia, *SAS* Zung Self-Rating Anxiety Scale, *SDS* Zung Self-Rating Depression Scale; *HRV* heart rate variability

The study will adhere to the declaration of Helsinki principles (World Medical Association version, 2013) [23]. All participants will be asked to sign the informed consent form before enrollment in this trial. The protocol has already been ethically reviewed and approved by the Sichuan Regional Ethics Review Committee on Traditional Chinese Medicine (ID:2021KL-059) in July 2021 (Supplementary file 1) and registered on the Chinese Clinical Trial Registry (https://www.chictr.org.cn/) (ID: ChiCTR2100049496). The study will follow the Standard Protocol Items: Recommendations for Interventional Trials (SPIRIT) guidance for protocol reporting (Supplementary file 2) [24].

### Participants

#### Recruitment

The FD patients will be recruited from the outpatient department of the Hospital of Chengdu University of Traditional Chinese Medicine (TCM) and the campus of the Chengdu University of TCM from September 2021 to December 2022. WeChat, posters, leaflets, free medical consultations, etc., will be used to inform and recruit potential FD patients. All potential FD patients will undergo a physical examination and laboratory tests, including normal gastroduodenoscopy, upper abdominal ultrasound, routine blood, urine and stool test, and blood biochemical test (ALT, AST, BUN, and Scr, etc.). A qualified digestion physician will make the final diagnosis for each potential FD patient.

#### Inclusion criteria

Patients will be enrolled if they meet all the following criteria: (1) right-handed and aged 18 to 40 years, (2) matching the Rome IV diagnosis criteria for FD (Supplementary file 3), (3) having no gastroduodenal structural diseases after gastroduodenoscopy screening, (4) having not taken any gastrointestinal prokinetic drugs at least 15 days before enrollment, (5) having not involved in other clinical trials, (6) having not any contraindications to MRI scan, and (7) signing an informed-consent form voluntarily (participants or their immediate family members).

#### Exclusion criteria

The patient will be excluded if they meet any of the following criteria: (1) being unconscious or being unable to cooperate in assessment; (2) suffering from cardiovascular, neurological, renal, liver, or blood diseases; (3) women being pregnant or lactating; (4) suffering from mental disorders such as anxiety or depressive disorder, bipolar disorder, schizophrenia, and claustrophobic syndrome; (5) having a history of head trauma, migraine, or dysmenorrhea; and (6) having received acupuncture or moxibustion treatment in the past month.

### Allocation and randomization

Eligible patients will be randomly allocated in a 1:1 ratio to the acupuncture or moxibustion group. An independent researcher will generate the random sequence by PASS 15.0 (NCSS, LLC. Kaysville, Utah, USA). Every random sequence number will be sealed in an opaque envelope by another research coordinator. The acupuncturists will finally open the sealed envelope to learn the group allocation of a participant prior to the delivery of the initial intervention session.

### Blinding

It is difficult to blind acupuncturists and patients in this trial for different types of intervention. Acupuncturists will not participate in any outcome assessment process. All the outcome assessors and statisticians will be masked in randomization assignments and intervention during the whole study.

### Withdrawal criteria and management

Participants will be withdrawn from the trial if (1) participants develop the serious disease, (2) seriously adverse events happen, and (3) participants request to withdraw from the trial, voluntarily. The reasons and exit time will be recorded in standard case report forms (CRFs). Patients who withdraw from the trial for any reason will be considered a drop-out. All the information from participants who have dropped out will be used for intention-to-treat (ITT) analysis.

### Sample size calculation

The sample size was estimated based on the results of a study on the changes in the short form Leeds Dyspepsia Questionnaire (SF-LDQ) in response to a 4-week acupuncture intervention versus moxibustion intervention, which showed a mean improvement of the SF-LDQ was − 5.62 ± 2.81 (mean ± SD) in the acupuncture group and − 3.56 ± 2.62 in the moxibustion group respectively [25]. It provides an effect size (Cohen's *d* = 0.758) to estimate the sample size of this study. To achieve 90% statistical power (*α* = 0.05), at least 76 participants are needed. Besides, assuming a 20% attrition rate, ninety-two participants (46 participants per group) will be recruited to detect a two-sided significant difference. The sample size calculation steps are shown in Supplementary file 4.

According to previous fMRI-based studies [26, 27], thirty subjects or more can notably improve the reliability of results. Considering the dropout rate or loss of data due to head motion during fMRI scanning, in this study, thirty-six participants in each group will be randomized to fMRI scans.

### Interventions

**Fig. 2:**
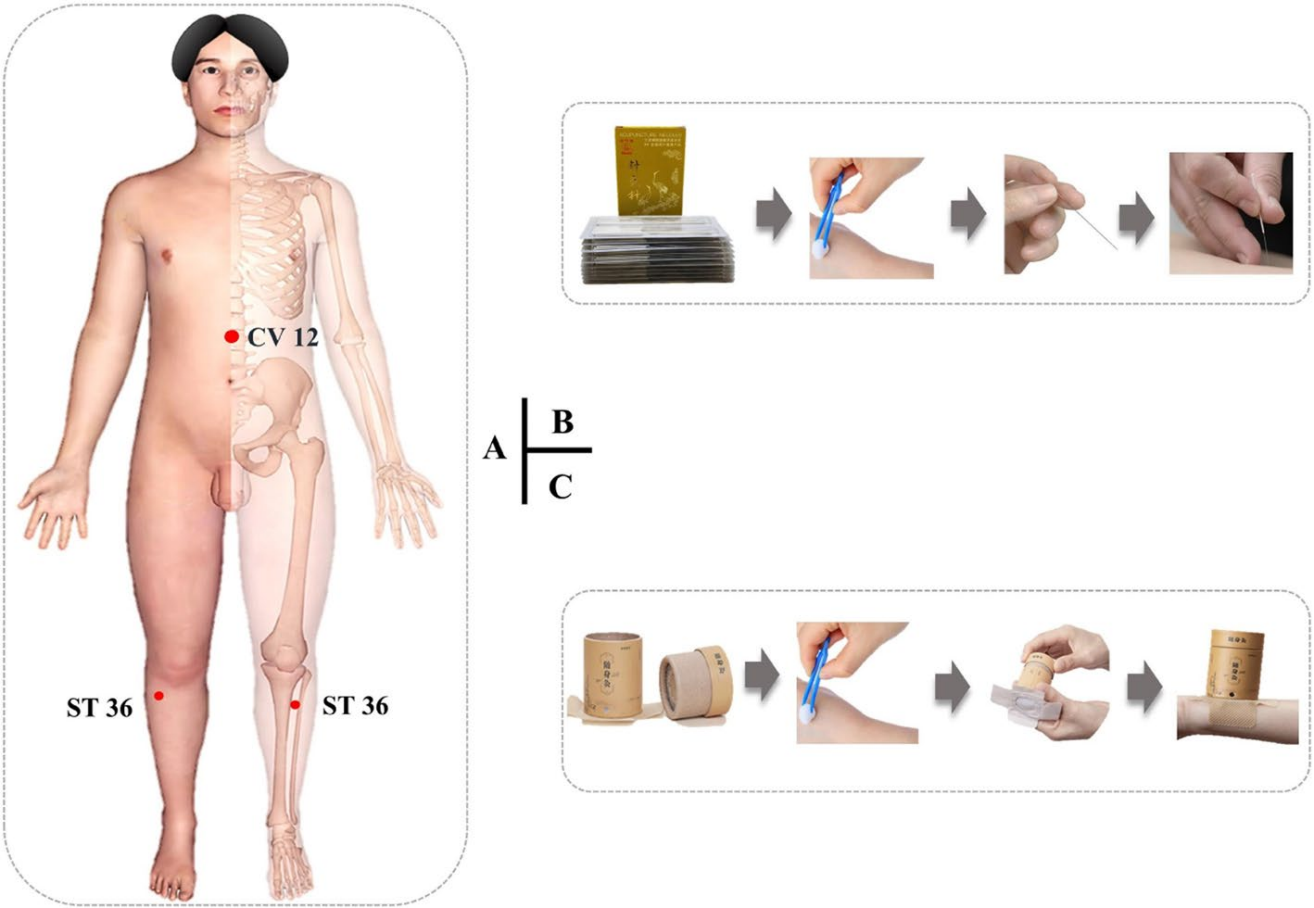
**A** Location of acupoints (CV-12 and ST-36). **B** *Hwato* acupuncture and brief acupuncture manipulation steps. **C** *Aikeshu* moxibustion stick and brief moxibustion manipulation steps

Participants in both groups will receive 20 sessions of acupuncture or moxibustion treatment in 4 weeks. Five times per week on *Zhongwan* (CV-12) and *Zusanli* (ST-36) will be selected as the treatment acupoints, which are the most commonly used acupoints for FD [28]. The locations of the acupoints are shown in Fig. 2A and Supplementary Table 1. All treatments will be performed by two acupuncturists. With at least 3 years of clinical experience and certified a 2-day standard training course.

#### Acupuncture intervention

After skin sterilization, the sterile needles (40 mm × 0.25 mm; Suzhou Medical Co., Ltd., China) (Fig. 2B) will be inserted perpendicularly into the acupoints (CV-12 and unilateral ST-36) with a depth of 25 to 30 mm. Then, the needle will be twisted between 90 and 180°, be lifted and thrust in an even amplitude between 3 and 5 mm, 60 times to 90 times per min to obtain the *deqi* sensation (*a special sensation of acupuncture*). The needles will be retained in the acupoints for 30 min, and the aforementioned manipulation will be applied every 10 min.

#### Moxibustion intervention

The lightened moxibustion sticks (13±1g; 60mm × 4.1mm; Bozhou Aikeshu Medical Co., Ltd., China) (Fig. 2C) will be placed on both CV-12 and unilateral ST-36. During the period of moxibustion, the temperature of the portable moxibustion will be adjusted according to the patient’s tolerance to prevent skin scald.

The *deqi* phenomenon is a special sensation of acupuncture and moxibustion, and it is closely related to clinical efficacy [29, 30]. The *deqi* sensation will be evaluated by the Chinese version of modified Massachusetts General Hospital Acupuncture Sensation Scale (C-MASS) [31], and the *deqi* sensation of moxibustion also will be evaluated by the C-MASS-based 10-point visual analog scale (VAS) form. Patients will self-assess the sensations by this scale. The sensation of acupuncture and moxibustion will be measured after the 1st and 20th interventions, respectively.

#### Concomitant treatments

If the symptoms are unbearable and FD patients have to take drugs (i.e., *Gastrointestinal motility regulating drugs*, *proton pump inhibitors*, *Chinese medicine*, etc.), participants need to tell us the time to take, dosage, and the specific information about the drugs, or any other therapy. Finally, we will record the details of the treatment in the case report form.

### Outcome measurements

The outcome measurements including (Table 1) the SF-LDQ, the Nepean Dyspepsia Index (NDI), the Symptom Index of Dyspepsia (SID), the Zung Self-Rating Anxiety Scale (SAS), and the Zung Self-Rating Depression Scale (SDS). Moreover, the evaluation time of these measurements was presented in the Table 1.

#### Primary outcome measurements

Our primary outcome is the improvement of SF-LDQ after 4 weeks of treatment. The SF-LDQ is a validated and reliable tool to measure the frequency and severity of five dyspeptic symptoms (epigastric pain, postprandial fullness, indigestion, epigastric burning, and postprandial nausea) [32]. Graded on a 5-point Likert scale, the higher the score of the five symptoms, the more severe the disorder.

#### Secondary outcome measurements

The secondary outcomes contain the NDI, the SID score, the SAS, and the SDS. Among them, NDI is a commonly used questionnaire to assess upper gastrointestinal symptoms and QoL of patients [33]. It includes two parts: symptom checklist (NDSI) and QoL questionnaire (NDQLI). A higher score of NDI indicates milder symptoms and better QoL. SID focuses on the 4 chief symptoms of FD (epigastric pain, burning, postprandial fullness, and early satiety) [34]. Using a 4-point Likert scale, the dyspeptic symptoms will be graded from 0 (none) to 3 (sever), and the total score will be used to evaluate the severity of dyspepsia [35]. The SAS and the SDS will be selected to evaluate the psychosocial state of FD patients [36, 37].

#### Heart rate variability test

The autonomic nervous system (ANS) plays a role in the physical function and pathological change of the digestive tract [38]. Dysfunction of the ANS may be an essential factor for the development of FD [39]. Heart rate variability (HRV) is a sensitive, quantitative, and intuitive indicator for the non-invasive assessment of autonomic nervous activity [40]. The 24-h dynamic electrocardiogram (ECG) (CT-086S, BENEWARE Co., Ltd., China) will be used to assess the HRV of patients. The metrics of HRV include the standard deviation of NN intervals (SDNN), the standard deviation of sequential 5-min RR interval means (SDANN), and the root mean square successive difference (RMSSD), etc. HRV will be measured after randomization and the 20th treatment.

### MRI scan

The fMRI data will be acquired with the Siemens 3.0T MRI (Siemens, Munich, Germany) at the Imaging Center of the fifth Chengdu Hospital, Sichuan, China. A 3-dimensional MRI sequence will be used to gain the high-resolution structural image. The parameters as following: repetition time/echo time = 1900 ms/2.26 ms, slices = 176, data matrix = 256 × 256, field of view = 256 × 256 mm^2^, and slice thickness = 1 mm. The parameters of blood oxygenation level-dependent resting-state functional image will be set as follows: repetition time/echo time = 2000 ms/30 ms, flip angle = 90°, slices = 30; data matrix = 64 × 64, field of view = 240 × 240 mm^2^, slice thickness = 5 mm, total volume = 240, and total scan time = 480 s. Twenty-four hours before scanning, every patient will be asked to keep their regular lifestyle, avoid staying up late, smoking, and drinking coffee or tea. Before scanning, every patient will stay at a restroom for 30 min and keep calm without thinking. During scanning, patients will be required to keep their eyes closed and ears plugged.

### Patient safety

Safety monitoring will be executed strictly throughout the study with reporting for adverse events (AEs) and serious adverse events (SAEs) such as subcutaneous hemorrhage, vertigo, infection, and burn and scaled injures. AEs/SAEs will be handled properly, recorded carefully, and report the SAEs and treatment to the principal investigator and the Ethics Committee immediately. We will pay all medical fees. Additionally, free medical and psychological consultation will be provided until they recover from AEs/SAEs.

### Data management

Clinical data will be managed with printed case report forms and electronic data capture (EDC). An independent data administrator will keep the data. Only principal investigators have the access to the full dataset and will perform double-data entry. The evidence-based medicine center of the CDUTCM is responsible for monitoring the study and data every 3 months and will make the final decision to terminate the trial. We will publish our results in peer-reviewed journals.

### Data analysis

#### Clinical data analysis

All analyses will be done on the ITT population. An independent statistician will conduct clinical data analysis with SPSS 24.0 software (SPSS Inc., Chicago, IL, USA). Kolmogorov–Smirnov test will be used to analyze data distribution, and Levene’s test will be used to analyze homogeneity of variance. The quantitative variables will be presented as mean and SD or median and interquartile, whereas qualitative measures will be reported as percentages with 95% CI. Analysis of variance (ANOVA) test will be used with Bonferroni post hoc comparisons for continuous variables. The independent-samples *t*-test or the Mann-Whitney *U* test for continuous variables and the chi-square test, Fisher exact test, or Kruskal–Wallis test for categorical variables will be used, when suitable. We will describe 24-h HRV using time-domain measurements. Time-domain indices of HRV quantify the amount of variability in measurements of the interbeat interval (IBI), which is the time period between successive heartbeats. These values may be expressed in original units or as the natural logarithm (Ln) of original units to achieve a more normal distribution [41]. Pearson's coefficient will be used for bivariate correlations. All the statistical significance threshold tests will be set to *p*<0.05 with a two-tailed test.

#### fMRI data analysis

The fMRI data will be analyzed with MATLAB 2 017b (MathWorks Inc., Natick, MA, USA). After data preprocessing, amplitude of low-frequency fluctuation (ALFF), functional connectivity (FC), and large-scale functional brain network analysis, etc. will be conducted to investigate the difference of central responses between acupuncture and moxibustion. In voxel-based analysis, the threshold will be set to *p*<0.01 in voxel-level and *p*<0.05 with false discovery rate in cluster-level. While in connectome-based analysis, the threshold will be set to *p*<0.05 with false discovery rate corrected. Furthermore, the Pearson correlation analysis between the clinical results and fMRI results will be conducted to investigate the potential correlation between symptom improvements and brain activity changes elicited by different interventions.

## Discussion

This randomized control fMRI trial will compare the differences in clinical effects and the central responses between acupuncture and moxibustion. The results might deepen our understanding of the advantages and central mechanisms of acupuncture and moxibustion for FD and besides provide a reference for a better selection of interventions for treating FD.

### The similarities and differences between acupuncture and moxibustion

Acupuncture and moxibustion are two important external therapeutic methods in traditional Chinese medicine (TCM). Both of them are guided by meridian and acupoint theory, but their manipulation is different. Acupuncture is a kind of mechanical stimulation with inserting specific needles into the acupoints for lifting, thrusting, and twisting, while moxibustion is to place moxa or moxa stick directly or indirectly on an acupoint to produce a warm stimulation; Therefore, they are different and related in clinical practice. For example, a multicenter RCT showed that 4 weeks of acupuncture can significantly increase patient-reported adequate relief and improve FD symptoms and QoL, and the effects of acupuncture persisted through the 12-week follow-up without symptom relapse or rebound [11]. Besides, some trials demonstrated that moxibustion can also relieve dyspeptic symptoms and improve QoL of FD patients, as well as has a significantly instant regulating effect on the abnormal electrogastrogram (EGG) rhythm of FD patients [42, 43]. However, what are the advantages of acupuncture and moxibustion in improving symptoms and QoL? Are the underlying mechanisms of acupuncture and moxibustion different and in what ways? These issues remain unclear and worthy of investigation. To explore the therapeutic advantages of acupuncture and moxibustion for FD, this study selected five questionaries to evaluate FD patients from three aspects. First is using SF-LDQ, SID, and NDSI to assess the symptom improvement. Second is using NDQLI to evaluate the QoL. Third is investigating the psychological status by SAS and SDS.

To explore the similarities and differences in potential mechanism, the MRI  scans will be performed on the selsected patients in each group, and the HRV tests will be performed on all the patients, at the baseline and the end of the treatment. Using 24-h dynamic ECG, the HRV changes of FD patients in each group will be calculated to investigate the influence of acupuncture and moxibustion on the function of the autonomic nerve. By MRI scan, the cerebral activity changes will be analyzed to explore the brain responses to acupuncture and moxibustion, respectively. In addition, by correlation analysis, the correlations among HRV changes, cerebral activity alteration, and clinical variables improvement in each group will be used to further explore the characteristics in the pattern of central responses to acupuncture or moxibustion.

### Quality control approaches are the precondition for the result reliability

To improve the reliability of the results, strict quality control will be conducted in the following aspects: (i) the selection of FD patients. To avoid the influence of age, and handedness on cerebral function and structure, right-handed and aged 18 to 40 years patients will be recruited. (ii) Acupuncture and moxibustion manipulation. Two experienced registered Chinese Medicine acupuncturists will perform the manipulation with a standard operating procedure and the *deqi* sensation of acupuncture and moxibustion will be recorded. Furthermore, to quantify and control the dosage of moxibustion, a novel portable moxibustion stick (13±1g; 60mm × 4.1mm; Bozhou Aikeshu Medical Co., Ltd., China) will be used in this study, which can be easily fixed on an acupoint to burn for 30 min with a relatively stable temperature. (iii) MRI scan. All MRI scans will be performed in the morning by the same operator, and female patients will be performed during the same menstrual cycle. Twenty-four hours before scanning, participants will be asked to maintain their routine lifestyle, avoid staying up late, smoking, and drinking coffee or tea. Before being scanned, every patient will stay at a restroom for 30 min and keep calm without thinking. During scanning, patients will be required to keep their eyes closed and their ears plugged. To eliminate head motion, the participant’s head will be placed in the head mask, and a sponge will be used to strengthen the fixation of the head.

In summary, acupuncture and moxibustion are safe and effective for FD. However, the characteristics of these two therapies for treating FD remain uncertain. With the development of neuroimaging technology and analytical methods, it is possible to further investigate the advantages and the underlying mechanism. The results will provide a new perspective to understand the different clinical values of acupuncture and moxibustion for FD, and their central mechanism.

## Trial status

Currently, the trial is not yet in the process of patient recruitment. We plan to start patient recruitment in August 2021 and end in October 2022.

## Supplementary Information

**Additional file 1.** Ethics approval.

**Additional file 2.** SPIRIT checklist.

**Additional file 3.** Rome IV diagnosis criteria.

**Additional file 4.** Sample size calculation steps.

**Additional file 5: Table S1.** Detailed location of acupoints.

**Additional file 6.** Consent form.

## Abbreviations

FD: Functional dyspepsia
FGIDs: Functional gastrointestinal disorders
SF-LDQ: Short form Leeds Dyspepsia Questionnaire
NDI: Nepean Dyspepsia Index
SID: Severity Index of Dyspepsia
SAS: Zung Self-Rating Anxiety Scale
SDS: Zung Self-Rating Depression Scale
HRV: Heart rate variability
fMRI: Functional magnetic resonance imaging
