# Supplementary figures and images for: Therapeutic effects and central mechanism of acupuncture and moxibustion for treating functional dyspepsia: study protocol for an fMRI-based randomized controlled trial
Source: Trials. 2022 Jun 6;23:462. doi: 10.1186/s13063-022-06411-9 (PMC9169350; doi:10.1186/s13063-022-06411-9)

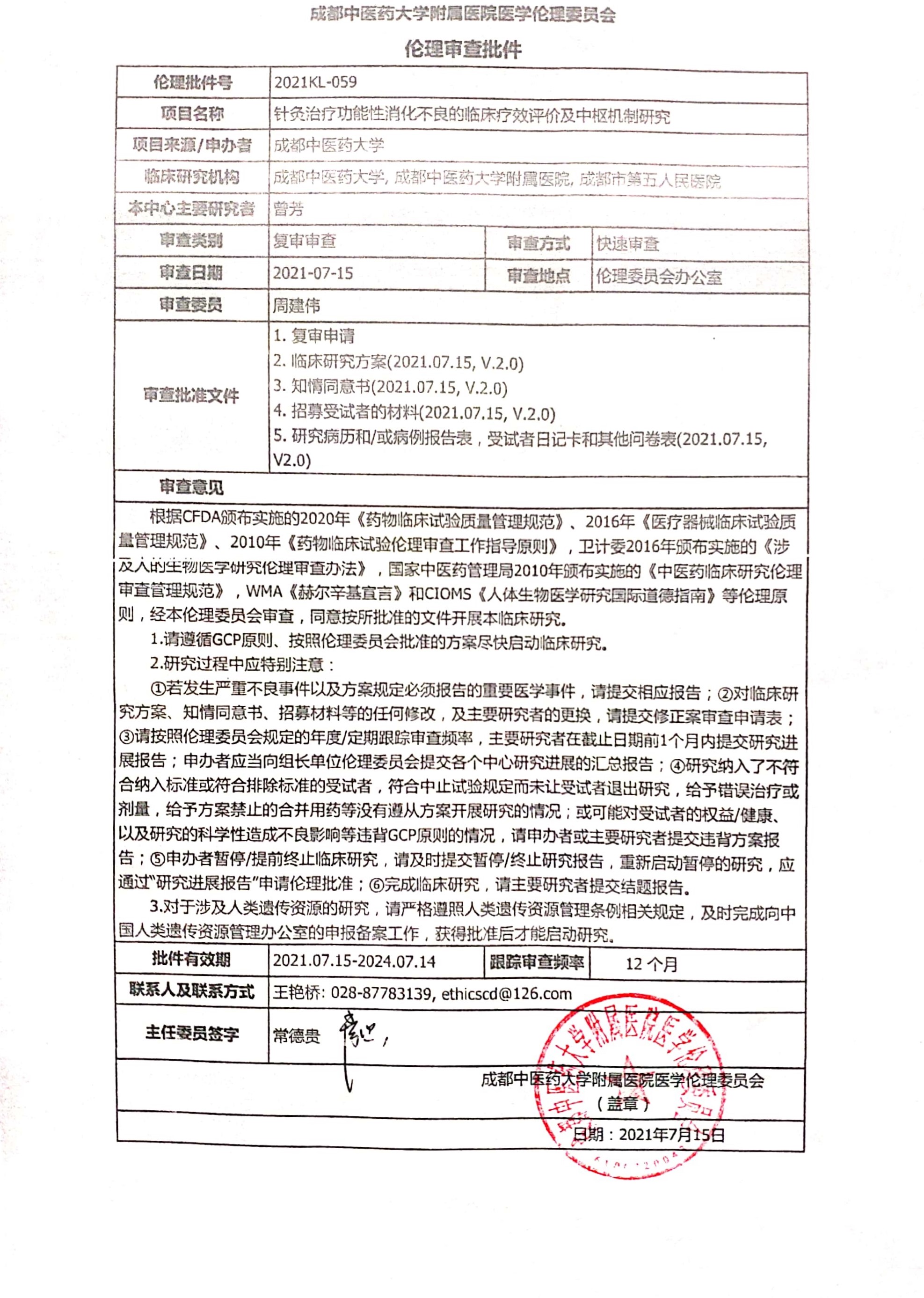

Supplement: Supplementary file 1 — Additional file 1. Ethics approval. [file 13063_2022_6411_MOESM1_ESM.jpg]
