# Supplementary material for: Therapeutic effects and central mechanism of acupuncture and moxibustion for treating functional dyspepsia: study protocol for an fMRI-based randomized controlled trial
Source: Trials. 2022 Jun 6;23:462. doi: 10.1186/s13063-022-06411-9 (PMC9169350; doi:10.1186/s13063-022-06411-9)
Supplement: Supplementary file 3 — Additional file 3. Rome IV diagnosis criteria. [file 13063_2022_6411_MOESM3_ESM.docx]

**Definition of Functional Dyspepsia According to the Rome IV Criteria**

According to the revised Rome IV criteria, functional dyspepsia is officially defined by:

i) Persistent or recurring dyspepsia for more than 3 months within the past 6 months;

ii) No demonstration of a possible organic cause of the symptoms on endoscopy;

iii) No sign that the dyspepsia is relieved only by defecation or of an association with stool irregularities.

In the current Rome IV criteria, 2 subgroups of functional dyspepsia are divided according to the cardinal symptoms (**Figure S1.**):

i) Epigastric pain syndrome (EPS)—predominant epigastric pain or burning;

ii) Postprandial distress syndrome (PDS)—feeling of fullness and early satiation.


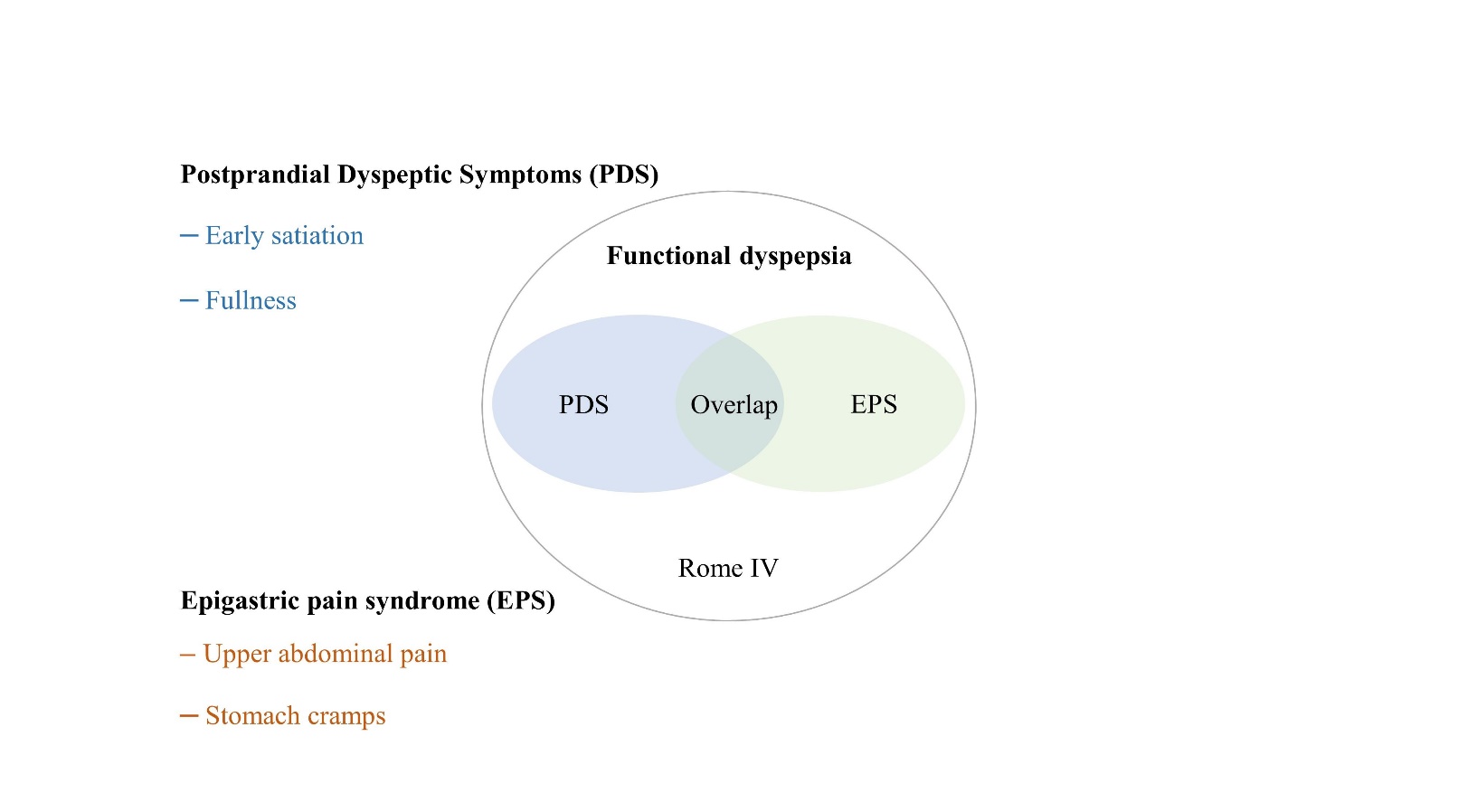


**Figure S1.**
