# Supplementary material for: Therapeutic effects and central mechanism of acupuncture and moxibustion for treating functional dyspepsia: study protocol for an fMRI-based randomized controlled trial
Source: Trials. 2022 Jun 6;23:462. doi: 10.1186/s13063-022-06411-9 (PMC9169350; doi:10.1186/s13063-022-06411-9)
Supplement: Supplementary file 4 — Additional file 4. Sample size calculation steps. [file 13063_2022_6411_MOESM4_ESM.docx]

**Brief steps and formulas for sample size calculation** [1,2]

***Step 1.***

To calculate the pooled standard deviation of **group 1** and **group 2**.

（1）

$$\boldsymbol{S}\mathbf{=}\sqrt{\frac{\left( \boldsymbol{n}_{\boldsymbol{1}}\boldsymbol{-1} \right)\boldsymbol{S}_{\boldsymbol{1}}^{\boldsymbol{2}}\boldsymbol{+}\left( \boldsymbol{n}_{\boldsymbol{2}}\boldsymbol{-1} \right)\boldsymbol{S}_{\boldsymbol{2}}^{\boldsymbol{2}}}{\boldsymbol{n}_{\boldsymbol{1}}\boldsymbol{+}\boldsymbol{n}_{\boldsymbol{2}}\boldsymbol{-2}}}$$

$\boldsymbol{n}_{\boldsymbol{1}}$:the actual sample size of group 1; $\boldsymbol{n}_{\boldsymbol{2}}:$ the actual sample size of group 2; $\boldsymbol{S}_{\boldsymbol{1}}$: the standard deviation of group 1; $\boldsymbol{S}_{\boldsymbol{2}}$：the standard deviation of group 2.

After calculation, we calculated that $\boldsymbol{S\approx}2.72$

***Step 2.***

To estimate the sample size of $\boldsymbol{n}_{\boldsymbol{1}}$ and $\boldsymbol{n}_{\boldsymbol{2}}$ based on the pooled standard deviation outcome aforementioned. With a 90% statistical power (α = 0.05). Besides, the sample size of the 2 groups is equal. We can get that $\boldsymbol{\mu}_{\boldsymbol{1-}\frac{\boldsymbol{\alpha}}{\boldsymbol{2}}}$=1.960, $\boldsymbol{\mu}_{\boldsymbol{1-}\boldsymbol{\beta}}$=1.282， and *k*=1. In addition, we expected that $\boldsymbol{\mu}_{\boldsymbol{1}}\boldsymbol{-}\boldsymbol{\mu}_{\boldsymbol{2}}\boldsymbol{=}2.1$.

（2）

$$\boldsymbol{n}_{\boldsymbol{1}}\boldsymbol{=}\boldsymbol{n}_{\boldsymbol{2}}\boldsymbol{=}\frac{{\boldsymbol{(}\boldsymbol{\mu}_{\boldsymbol{1-}\frac{\boldsymbol{\alpha}}{\boldsymbol{2}}}\boldsymbol{+}\boldsymbol{\mu}_{\boldsymbol{1-}\boldsymbol{\beta}}\boldsymbol{)}}^{\boldsymbol{2}}\boldsymbol{\times}\boldsymbol{S}^{\boldsymbol{2}}\boldsymbol{\times(1+}\frac{\boldsymbol{1}}{\boldsymbol{k}}\boldsymbol{)}}{{\boldsymbol{（}\boldsymbol{\mu}_{\boldsymbol{1}}\boldsymbol{-}\boldsymbol{\mu}_{\boldsymbol{2}}\boldsymbol{）}}^{\boldsymbol{2}}}$$

***S***: combined standard deviation; $\boldsymbol{\mu}_{\mathbf{1}-\frac{\boldsymbol{\alpha}}{\mathbf{2}}}$: standard normal variate at 0.05 type I error;$\boldsymbol{\mu}_{\mathbf{1}-\boldsymbol{\beta}}$: standard normal variate for power; $\boldsymbol{\mu}_{\boldsymbol{1}}$: mean value of group 1; $\boldsymbol{\mu}_{\boldsymbol{2}}\boldsymbol{-}\boldsymbol{\mu}_{\boldsymbol{2}}$: effect size or difference in proportion expected based on previous studies; ***k***: ratio of control to cases, 1 for the equal number of case and control.

Finally, we calculated that $\boldsymbol{n}_{\boldsymbol{1}}\boldsymbol{=}\boldsymbol{n}_{\boldsymbol{2}}\boldsymbol{=}38$

**References**

1 Chow S-C, Wang H, Shao J. *Sample size calculations in clinical research*. Chapman and Hall/CRC 2007.

2 Charan J, Biswas T. How to calculate sample size for different study designs in medical research? *Indian J Psychol Med* 2013;**35**:121. doi:10.4103/0253-7176.116232
