# Supplementary material for: Therapeutic effects and central mechanism of acupuncture and moxibustion for treating functional dyspepsia: study protocol for an fMRI-based randomized controlled trial
Source: Trials. 2022 Jun 6;23:462. doi: 10.1186/s13063-022-06411-9 (PMC9169350; doi:10.1186/s13063-022-06411-9)
Supplement: Supplementary file 5 — Additional file 5: Table S1. Detailed location of acupoints. [file 13063_2022_6411_MOESM5_ESM.docx]

**Detailed location of acupoints**

| **Acupoints** | **Location** |
| --- | --- |
| *Zhongwan (CV-12)* | On the anterior median line of the upper abdomen, 4 *cun** above the navel. |
| *Zusanli (ST-36)* | On the anterior side of side of the shank, 3 *cun** below Dubi (ST-35), one horizontally-placed figure distance lateral to the anterior border of the tiba (The middle finger). |

*cun**：Chinese unit of length，one cun is the distance between the ends of the interphalangle creases of the patients’ middle figure or the greatest width of the distal phalanx of the thumb.
