# Supplementary material for: Therapeutic effects and central mechanism of acupuncture and moxibustion for treating functional dyspepsia: study protocol for an fMRI-based randomized controlled trial
Source: Trials. 2022 Jun 6;23:462. doi: 10.1186/s13063-022-06411-9 (PMC9169350; doi:10.1186/s13063-022-06411-9)
Supplement: Supplementary file 6 — Additional file 6. Consent form. [file 13063_2022_6411_MOESM6_ESM.doc]

**知情同意书·知情告知页**

尊敬的患者：

医生已经确诊您患有功能性消化不良，我们诚邀您参加由成都中医药大学“杏林学者”学科人才科研提升计划项目（NO. RCPT2018001）支持的“针灸治疗功能性消化不良的临床疗效评价及中枢机制研究”，该研究方案已通过四川省中医药区域伦理委员会的审查（NO. 2021KL-059）。

在您决定是否参加这项研究之前，请尽可能仔细阅读以下内容。它可以帮助您了解该项研究以及为何要进行这项研究，研究的程序和期限，参加研究后可能给您带来的益处、风险和不适。如果您愿意，您也可以和您的亲属、朋友一起讨论，或者请医生给予解释，帮助您做出决定。

**一、研究背景和研究目的**

功能性消化不良（Functional dyspepsia, FD）是临床最为常见的消化系统疾病之一。目前，FD以其发病率高、社会经济负担重以及对生活质量影响显著而成为重要的社会公共卫生问题。国外文献报道FD的发病率为20%～40%，国内统计约为23.5%，占国内消化内科门诊患者数的40%左右。

针灸治疗本病历史悠久、疗效显著。前期临床研究结果显示，针灸治疗能有效改善患者临床症状，显著提高患者生活质量，但针灸治疗FD效应机制仍有待进一步研究。基于此，本研究将在评价针灸治疗FD临床疗效的基础上，从脑网络整合角度入手，揭示针灸治疗FD的中枢机制异同。

本试验将于2021年7月至2023年12月，在成都中医药大学针灸推拿学院、成都中医药大学附属医院消化内科、成都市第五人民医院进行，共有92位受试者参加。

**二、哪些人适宜参加本研究**

☆ 右利手，年龄18岁≤年龄≤40岁，大专及以上学历；

☆ 符合FD的罗马IV诊断标准；

☆ 经胃镜检查，无局部器质性病变；

☆ *Hp*检查阴性，或*Hp*阳性根除治疗后症状仍然存在；

☆ 近15天内没有服用过任何胃肠促动力药物，且未参与其他临床研究；

☆ 患者本人签署或其直系亲属代签知情同意书。

**三、哪些人不宜参加研究**

☆ 意识不清，不能表达主观不适症状者及精神病患者；

☆ 合并心血管、肝、肾、消化、血液系统等严重原发性疾病者；

☆ 孕妇、哺乳期妇女及近半年内有妊娠计划者；

☆ 伴有严重的抑郁、焦虑症状者；

☆ 伴有严重痛经症状的女性患者；

☆ 伴有明显的头痛、偏头痛、痛经，头部外伤史者；

☆ 伴有幽闭恐惧症、体内金属移植、纹身等磁共振扫描禁忌症者。

**四、如果参加研究将需要做什么？**

1. 在您入选研究前，您将接受以下检查以确定您是否可以参加研究：

（1）医生将询问、记录您的病史，并进行体格检查。

（2）进行普通胃镜、腹部B超、24小时动态心电图、肝肾功能、三大常规检查。

2. 若您是筛查符合纳入标准的FD患者，将按以下步骤进行研究。

（1）医生首先将根据计算机提供的随机数字，决定您接受何种治疗。您分别有1/2的可能性被分入针刺组与艾灸组中的任何一个，但每种治疗方式对FD而言都是有效的，以上分组，您和您的医生都无法事先知道和选择。

（2）您将有可能在针刺治疗前和治疗后接受2次功能磁共振（functional Magnetic Resonance Imaging，fMRI）扫描，每次fMRI检查大概需要30分钟，它是安全无辐射的检查方法，不会对您的健康造成损害。

（3）本研究每次治疗时间为30分钟，每日进行一次，5次为一个疗程，疗程间休息2天，共治疗4个疗程。

（4）在您第1次治疗前，4个疗程治疗结束后和治疗结束后4周随访时，医生将对您临床症状、情绪状况进行评价，请您如实向医生反映病情变化。

3. 需要您配合的其他事项：

（1）您应当积极配合医生，按时、规律地接受治疗，并真实地向医生反应您病情变化情况。

（2）在研究期间我们不建议您使用任何改善胃部症状和情绪状况的药物，如您确实需要进行其它治疗，请事先与您的医生取得联系。

（3）在fMRI扫描的前一天，您应当避免饮酒、喝茶、喝咖啡，避免进行剧烈运动，避免剧烈的情绪波动。如有上述情况发生，请及时与您的医生联系。

**五、参加研究可能的受益**

您将可能从本项研究中受益。此种受益包括您的病情有可能获得改善。本项研究也有可能帮助医生和研究人员进一步明确针刺和艾灸治疗FD的临床疗效和起效机制，以用于患有相似病情的其他病人。如果您按计划完成试验，将在将来的6个月内获得项目组提供的免费健康咨询服务。

尽管已经有证据提示本研究所采用的针灸疗法对FD的治疗作用，但这并不能保证对您肯定有效。本研究所采用的方法也并非FD治疗的唯一的方法，除针灸疗法以外，药物治疗(包括中药和西药治疗)、认知疗法也被证明对FD有效。您可以向医生询问有可能获得的替代治疗方法。

**六、参加研究可能的不良反应、风险和不适、不方便**

在治疗过程中可能会出现的晕针、皮下出血或烫伤等意外情况。当发生不良事件时，无论其是否与本研究治疗方法有关，均应及时通知您的医生，他/她将对此做出判断并给与适当的医疗处理。同时，研究者会进行详细记录，包括：不良事件与严重不良事件的发生时间、中止时间和持续时间（可以用天或h来记录），严重程度及频率，处理方法及结果，对不良事件与试验治疗方法因果关系的分析，不良事件与严重不良事件的跟踪情况等。有关不良事件的所有临床资料，如检查单据、处方等均应会保存在原始文件中。

**七、有关费用**

（1）您在成都市第五人民医院或成都中医药大学附属医院参加相关检查的费用（包括普通胃镜、腹部B超、24小时动态心电图、肝肾功能、三大常规检查和功能磁共振扫描）和治疗的费用将由项目组承担。

（2）前往医院检查所产生的路费根据实际情况报销。

（3）如果在临床试验中出现不良事件，医学专家委员会将会鉴定其是否与针刺有关。如不良事件为本研究治疗方式所引起，项目组将提供治疗的费用及相应的经济补偿。

（4）对于您同时合并的其他疾病所需的治疗和检查，将不在免费的范围之内。

（5）在您完成试验后项目组将不再给予额外的受试者补偿费。

**八、个人信息是保密的吗？**

您的医疗记录（研究病历/CRF、化验单等）将完整地保存在您所就诊的医院。医生会将化验检查结果记录在您的病历上。研究者、伦理委员会和医疗管理部门将被允许查阅您的医疗记录。任何有关本项研究结果的公开报告将不会披露您的个人身份。我们将在法律允许的范围内，尽一切努力保护您个人医疗资料的隐私。

**九、怎样获得更多的信息？**

您可以在任何时间提出有关本项研究的任何问题，并得到相应的解答。

如果在研究过程中有任何重要的新信息，可能影响您继续参加研究的意愿时，您的医生将会及时通知您。

**十、可以自愿选择参加研究和中途退出研究**

是否参加研究完全取决于您的意愿。您可以拒绝参加此项研究，或在研究过程中的任何时间退出，这都不会影响您和医生间的关系，不会造成您的医疗或其他方面利益的损失。

出于对您的最大利益考虑，医生或研究者可能会在研究过程中随时中止您继续参加本项研究。

如果您因为任何原因从研究中退出，您可能被询问有关您进行治疗的情况和结果。如果医生认为需要，您也可能被要求进行实验室检查和体格检查。

**十一、现在该做什么？**

是否参加本项研究由您自己（和您的家人）决定。

在您做出参加研究的决定前，请尽可能向你的医生询问有关问题。

感谢您阅读以上材料。如果您决定参加本项研究，请告诉您的医生，他/她会为您安排一切有关研究的事务。

**知情同意书·同意签字页**

**项目名称：**针灸治疗功能性消化不良的临床疗效评价及中枢机制研究

**课题承担单位：**成都中医药大学

**课题协作单位：**成都中医药大学附属医院、成都市第五人民医院

同意声明

我已经阅读了上述有关本研究的介绍，而且有机会就此项研究与医生讨论并提出问题，我提出的所有问题都得到了满意的答复。

我知道参加本研究可能产生的风险和受益。我知晓参加研究是自愿的，我确认已有充足时间对此进行考虑，而且明白：

- 我可以随时向医生咨询更多的信息。
- 我可以随时退出本研究，而不会受到歧视或报复，医疗待遇与权益不会受到影响。

我同样清楚，如果我中途退出研究时，我若将我的病情变化告诉医生，完成相应的体格检查和理化检查，这将对整个研究十分有利。

如果因病情变化我需要采取任何其他的药物治疗，我会在事先征求医生的意见，或在事后如实告诉医生。

我同意卫生管理部门、伦理委员会或申办者代表查阅我的研究资料。

我将获得一份经过签名并注明日期的知情同意书副本。

最后，我决定同意参加本项研究，并保证尽量遵从医嘱。

**患者签名： ＿ ＿ 年 ＿ ＿ 月 ＿ ＿ 日**

**联系电话（手机）：**

我确认已向患者解释了本试验的详细情况，包括其权利以及可能的受益和风险，并给其一份签署过的知情同意书副本。

**医生签名： ＿ ＿ 年 ＿ ＿ 月 ＿ ＿ 日**

**联系电话(手机)：**
